# Supplementary material for: SAMHD1 promotes SARS-CoV-2 infection by enhancing HNF1-dependent ACE2 expression in lung epithelial cells
Source: bioRxiv. 2025 Oct 26:2025.10.23.684143. Preprint. [Version 2] doi: 10.1101/2025.10.23.684143 (PMC12633470; doi:10.1101/2025.10.23.684143)

Supplementary Fig. S1

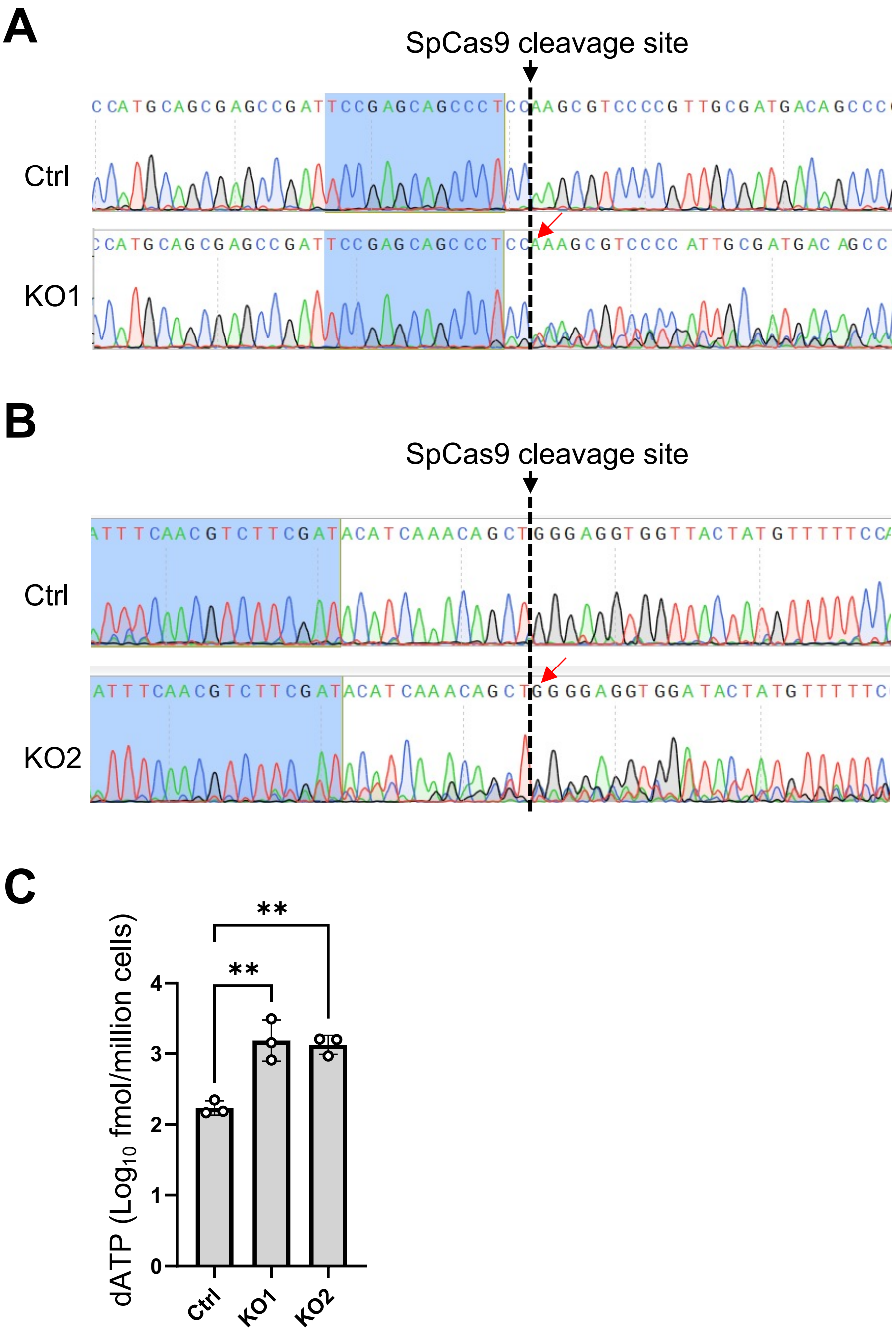

Supplementary Fig. S2

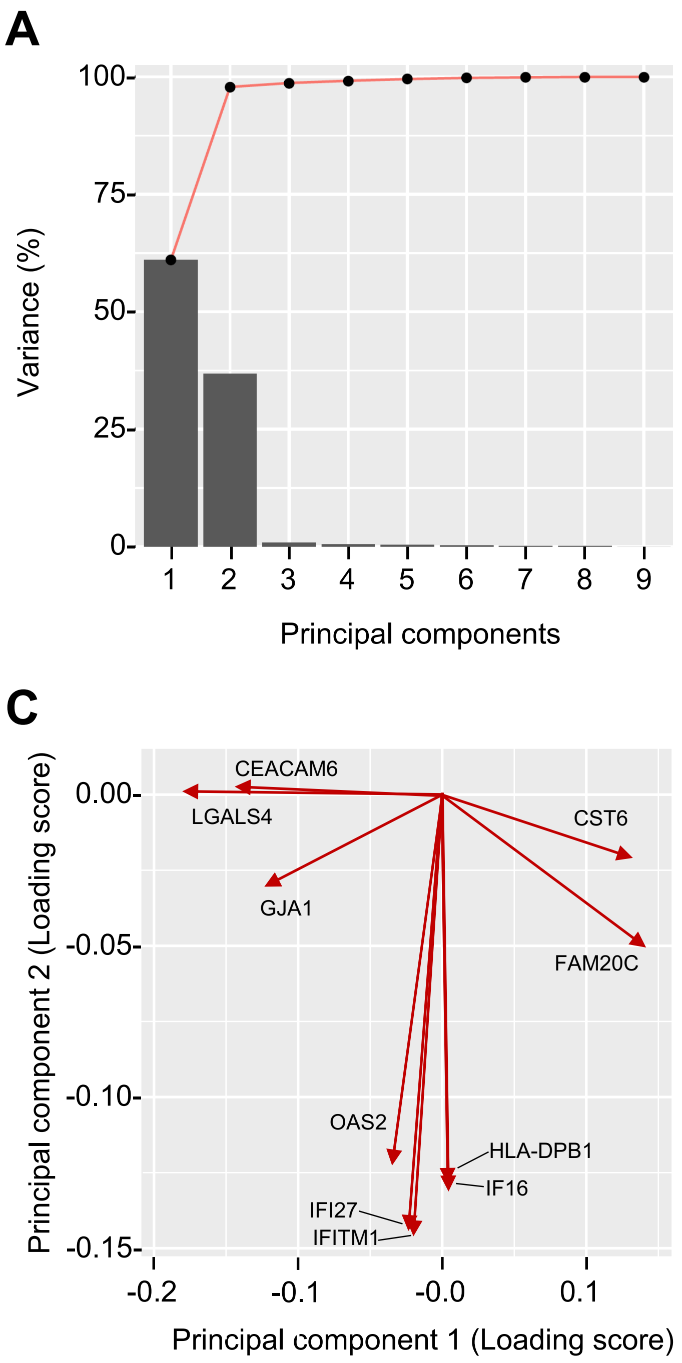

Supplementary Fig. S3

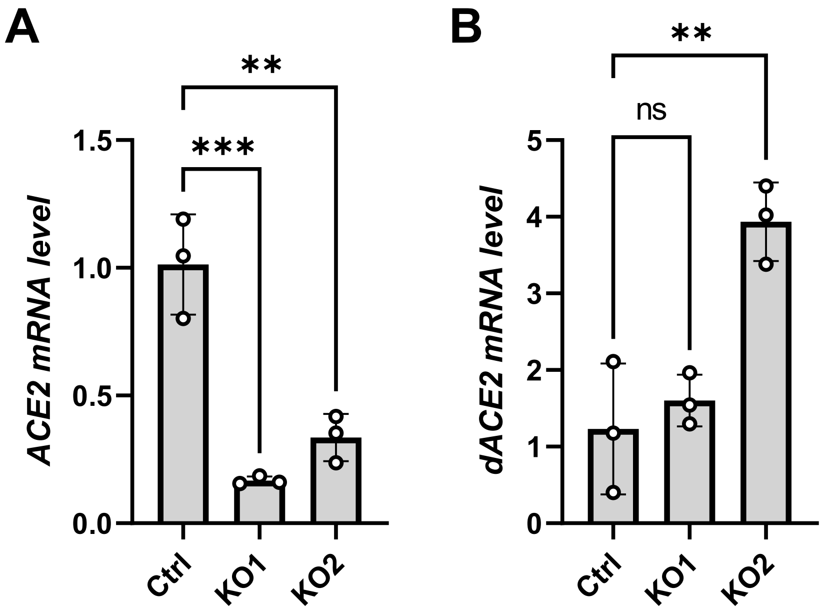

Supplementary Fig. S4

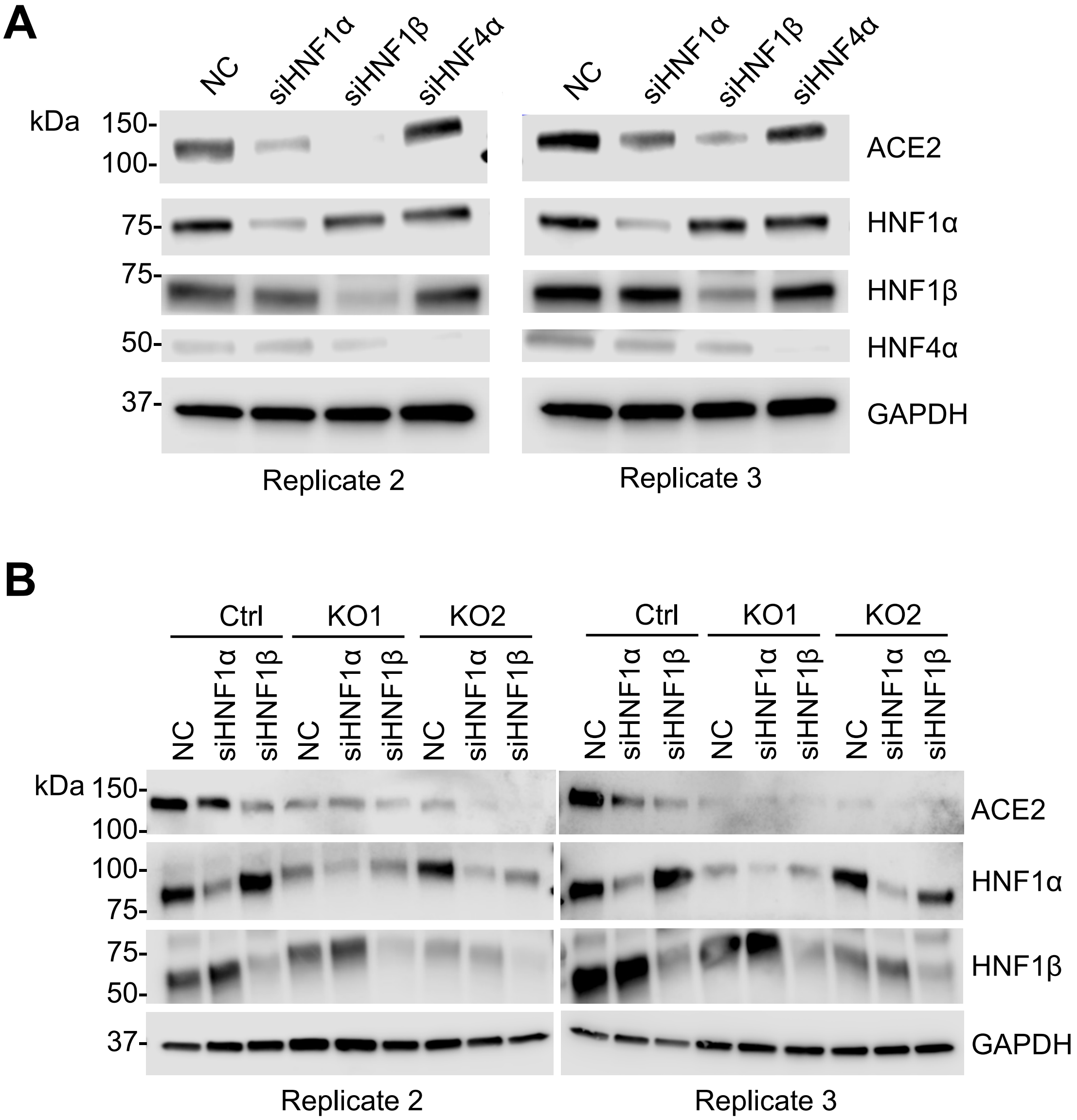

Supplementary Fig. S5

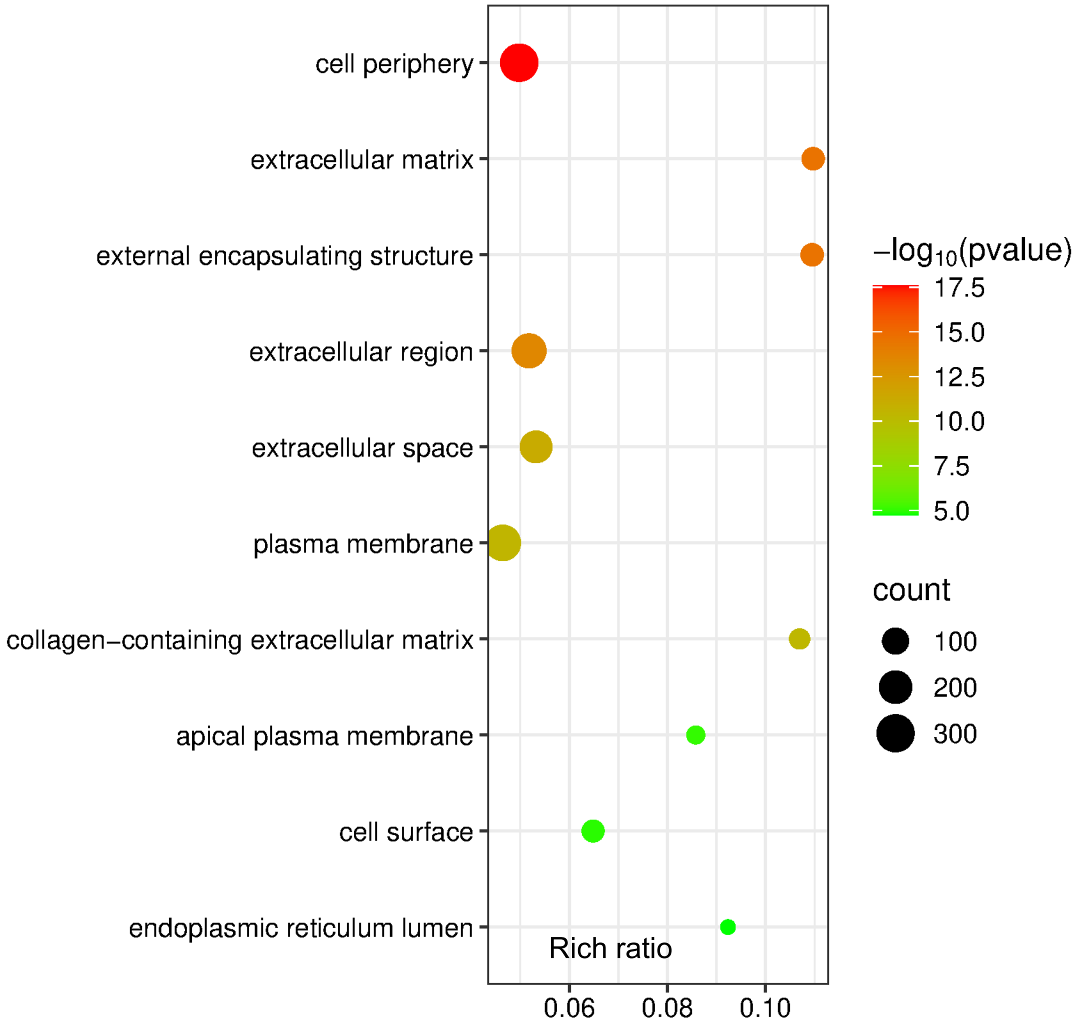

Supplement: 1 — Supplementary Fig. S1. Validation of SAMHD1 deficiency in SAMHD1 KO Calu-3 cells. (A and B) The sanger sequencing results to the regions of SpCas9 cleavage sites directed by respectively sgRNA1 or sgRNA2 of (A) KO1 and (B) KO2 cells were shown and compared with that of the Ctrl cells. Red arrows indicated one base pair insertion near the SpCas9 cleavage site. (C) dATP levels of Calu-3 Ctrl, KO1 or KO2 cells were measured with HIV-1 RT based single nucleotide incorporation assay. For (C), one-way ANOVA multiple comparisons test was used to evaluate the statistical significance of the difference between Ctrl and KO1 or KO2. ** P<0.01. ns, not significant. Supplementary Fig. S2. PCA clustering of Calu-3 Ctrl, KO1 and KO2 cells based on the mRNA-seq profiles. Nine samples of the mRNA-seq analysis gave nine PCs (i.e. Ctrl, KO1 and KO2 cells, biological triplicate). The influence of each PC was plotted based on their contributing variance (%). The cumulative variances from 1 to 9 were plotted above and joined by a curve. PC1 and PC2 gave 98% coverage of all PCs. (B) Individual samples from Ctrl, KO1 and KO2 cells were plotted along the PC1 and PC2 based on the net scores of loading genes obtained from PCA. (C) A PCA biplot was generated by top ten loading genes influencing PC1 and PC2. Supplementary Fig. S3. Confirming ACE2 and dACE2 mRNA expressions in SAMHD1 KO Calu-3 cells. The mRNA levels of (A) ACE2 and (B) dACE2 of Ctrl, KO1 and KO2 cells were measured by RT-qPCR with qPCR primer pairs previously described [55]. For ACE2, primer pair 4 listed in Table S5 was used. Biological triplicate experiment was performed. One-way ANOVA multiple comparisons test was used to evaluate the statistical significance of the difference between Ctrl and KO1 or KO2. ** P<0.01, *** P<0.001. ns, not significant. Supplementary Fig. S4. Independent experiments performed for protein band calculation of Fig. 7B and Fig. 8B–8D. The Western blot results to two more independent experiment replic [file NIHPP2025.10.23.684143V2-supplement-1.pdf]
